# Mind the gap: can we explain declining male reproductive health with known antiandrogens?

**DOI:** 10.1530/REP-13-0440

**Published:** 2014-04

**Authors:** Andreas Kortenkamp, Martin Scholze, Sibylle Ermler

**Affiliations:** Institute for the Environment, Brunel UniversityKingston Lane, Uxbridge, UB8 3PHUK

## Abstract

Several countries have experienced rises in cryptorchidisms, hypospadias and testicular germ cell cancer. The reasons for these trends are largely unknown, but Skakkebaek has proposed that these disorders form a testicular dysgenesis syndrome and can be traced to androgen insufficiency in foetal life. This suggests that antiandrogenic chemicals might contribute to risks, but few chemicals have been linked to these diseases in epidemiological studies. In animal studies with *p,p* *′*-dichlorodiphenyldichloroethylene, effects typical of disruptions of male sexual differentiation became apparent when the foetal levels of this androgen receptor (AR) antagonist approached values associated with responses in *in vitro* assays. This prompted us to analyse whether the 22 chemicals with AR antagonistic properties would produce mixture effects in an *in vitro* AR antagonism assay when combined at concentrations found in human serum. Other antiandrogenic modalities could not be considered. Two scenarios were investigated, one representative of average serum levels reported in European countries, the other in line with levels towards the high exposures. In both situations, the *in vitro* potency of the 22 selected AR antagonists was too low to produce combined AR antagonistic effects at the concentrations found in human serum, although the high exposure scenario came quite close to measurable effects. Nevertheless, our analysis exposes an explanation gap which can only be bridged by conjuring up as yet undiscovered high potency AR antagonists or, alternatively, high exposures to unknown agents of average potency.

## Introduction

In recent years, several countries have experienced increases in the incidence of cryptorchisms (reviewed by Main *et al*. (2010)) and hypospadias (Pierik *et al*. 2002, Boisen *et al*. 2005, Nelson *et al*. 2005, Nassar *et al*. 2007), the most frequent congenital malformations in young boys. The incidence of testicular germ cell cancers has risen steadily in Caucasian white men (Chia *et al*. 2010) and is now the most commonly diagnosed malignant neoplasm among men of 15–34 years of age.

There are clear regional differences, but factors that fully explain these trends and differences have remained elusive. Although alcohol consumption, low birth weight, premature birth and diets lacking in protein are recognised risk factors for cryptorchidism and hypospadias, these alone cannot account for the current disease trends. Skakkebaek *et al*. (2001) have proposed that cryptorchidisms and hypospadias are part of a syndrome that also comprises poor semen quality and testicular germ cell cancers, termed testicular dysgenesis syndrome (TDS). Changes in anogenital distance (AGD) are now also considered part of the TDS, since AGD is a biomarker of androgen action in foetal life that stays into adult life (Swan *et al*. 2005). The TDS hypothesis suggests that its component disorders arise from insufficient androgen action in foetal life and proposes that exposures to antiandrogenic chemicals are an aetiological factor. In this context, antiandrogenicity is commonly understood to include several modes of action, such as androgen receptor (AR) antagonism and suppression of foetal androgen synthesis, all resulting in androgen insufficiency.

Epidemiological studies demonstrating associations between antiandrogenic chemicals and TDS disorders are few and far between. This complicates an assessment of the TDS hypothesis, not least because only a limited range of chemicals have been investigated. Studies of paternal and maternal pesticide exposures in agricultural occupational settings have reported associations with cryptorchidisms and hypospadias; however, due to their design, they could not pinpoint specific chemicals (Pierik *et al*. 2004, Carbone *et al*. 2006). There is evidence for associations of diethylstilboestrol (DES) (Palmer *et al*. 2009) and polybrominated diphenyl ethers (Carmichael *et al*. 2010) with the risk of developing cryptorchidisms. Associations between testicular germ cell cancers and exposure to chlordanes, *p,p*-dichlorodiphenyldichloroethylene (*p,p*-DDE) and certain 3,3′,4,4′,5-pentachlorobiphenyls (PCBs) have been detected in several epidemiological studies, but the magnitude of effects was relatively small (Hardell *et al*. 2003, 2004, 2006, McGlynn *et al*. 2008). There are several reports of associations between phthalate exposure during pregnancy and changes in AGD (Swan *et al*. 2005, Bustamante-Montes *et al*. 2008, Swan 2008, Suzuki *et al*. 2012), but as yet there is no information whether phthalates are capable of contributing to the risk of developing cryptorchidisms, hypospadias or testis cancer. Other chemicals identified as antiandrogens in animal models or *in vitro* assays, such as certain azole pesticides, benzophenones, parabens or synthetic musks have not been investigated in epidemiological studies. Of the chemicals for which associations with TDS disorders have been noted, only certain polybrominated diphenyl ethers (Stoker *et al*. 2005) and phthalates (Wine *et al*. 1997) are recognised as antiandrogens.

The associations between TDS disorders and individual chemicals analysed in epidemiological studies are relatively weak, with odds ratios not far above 1. This suggests that the investigated chemicals, considered in isolation, do not make a strong contribution to health risks and that other, as yet unidentified, influences may be at play, including new and emerging chemicals not yet recognised as contributing to TDS disorders. Another aspect likely to increase risk estimates is the reality of combination effects between several chemical exposures, not addressed in the available epidemiological studies.

**Figure 1:**
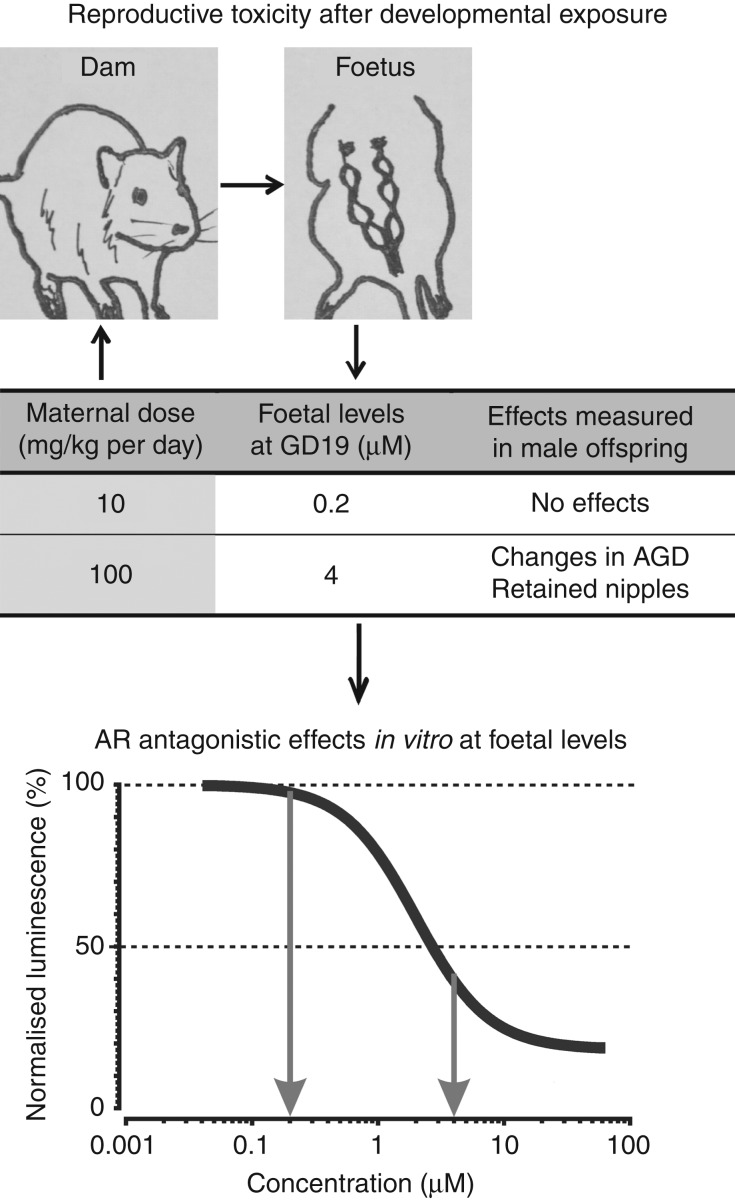
Illustration of the tissue dosimetric approach by You *et al*. (1999). Shown is how the foetal tissue concentrations of *p,p* *′*-DDE attained through maternal dosing relate to the *in vitro* AR antagonistic effects of *p,p* *′*-DDE measured by Orton *et al*. (2011), bottom graph. The two vertical arrows in the bottom graph are the two foetal *p,p* *′*-DDE tissue levels measured at gestational day 19. It can be seen that the lower maternal dose which was not associated with demasculinising effects did not give rise to *in vitro* AR antagonistic effects. In contrast, the higher maternal dose induced marked demasculinising effects and gave rise to foetal *p,p* *′*-DDE levels that produced 70% AR antagonistic effects *in vitro*.

Since antiandrogenicity is central to the TDS hypothesis, we wondered whether antiandrogens known to be present in human tissues are sufficiently potent, and present in sufficiently high levels and numbers to result in antiandrogenic effects. We combined tissue dosimetry with data sets describing *in vitro* AR antagonist potencies of chemicals and measurements of human tissue levels, and investigated a possible explanation gap from the perspective of mixture toxicology. A study of the AR antagonist *p,p*-DDE (You *et al*. 1999) in a developmental toxicity model in the rat inspired us to take this approach. To our knowledge, this is the only paper to date that has anchored antiandrogenic effects in rats to *p,p*-DDE tissue levels in the foetus. You *et al*. (1999) dosed pregnant dams with *p,p*-DDE (10 and 100 mg/kg per day) from gestational day (GD) 14 to 18, during the male programming window. Effects on landmarks of male sexual differentiation (changes in AGD and retained nipples) were only observed at the higher dose of 100 mg/kg per day. The concentrations of *p,p*-DDE measured in the male foetuses on GD15, 17 and 19 reached values between 2 and 7 μmol/l, a concentration range associated with 20–70% AR antagonism in *in vitro* AR antagonist assays based on MDA-kb2 cells (Wilson *et al*. 2002, Orton *et al*. 2011) or CHO-K1 cells (Kojima *et al*. 2004). The foetal *p,p*-DDE levels that resulted from the smaller dose of 10 mg/kg per day to the dams (0.2–0.35 μmol/l) were too low to produce *in vitro* AR antagonism and in fact did not elicit AGD changes or retained nipples in the study by You *et al*. (1999) (Fig. 1).

This interesting concordance led us to assess the magnitude of combined antiandrogenic effects that can be expected on the basis of published measurements of the human tissue levels of antiandrogenic chemicals and their *in vitro* potency. In approaching this issue, we could rely on extensive evidence that the joint effects of multi-component mixtures of *in vitro* AR antagonists (up to 30 components) can be approximated quite well by using the mixture assessment concept of dose addition (Birkhoj *et al*. 2004, Kjærstad *et al*. 2010, Ermler *et al*. 2011, Orton *et al*. 2012, 2013). According to the principles of dose addition, a concentration of *p,p* *′*-DDE associated with *in vitro* activity (and accordingly, a foetal concentration linked with *in vivo* effects) can be replaced with several equi-effective fractions of other active chemicals, without loss of effect. This opened the way for making an attempt of predicting the combined effects of antiandrogens in human tissues entirely by modelling, without conducting the actual mixture experiments.

However, the dose addition principle together with application of an *in vitro* AR antagonist assay meant that we had to restrict our analysis to AR antagonists. The possible contribution of antiandrogens that operate through other modes of action, for example phthalates, could not be taken into account directly, because such agents are without effect in *in vitro* AR antagonist assays. It is therefore not possible to integrate the effects of AR antagonists and those of chemicals that suppress foetal androgen synthesis at the level of AR antagonism. This can only be achieved by conducting *in vivo* studies, but considering that only a handful of chemicals have been evaluated *in vivo*, exclusive reliance on *in vivo* data would have severely limited the scope of our analysis. The exclusion of phthalates from the analysis will have to be reflected on carefully when it comes to assessing the implications of our observations (see ‘Discussion’ section).

To realise the aims of our study, we had to rely on chemicals for which concentration–response relationships for AR antagonism *in vitro* had been described in detail. At the same time, information about human serum or lipid levels had to be available for each substance. This restriction reduced the range of chemicals that could be considered for analysis, mainly because the number of agents identified as *in vitro* AR antagonists by far exceeds the number of chemicals for which tissue level data are also available. For example, quite a few pesticides were found to be active (Kojima *et al*. 2004, Vinggaard *et al*. 2008, Orton *et al*. 2011), but tissue levels are essentially unknown. We utilised our data base of in* vitro* AR antagonists (Ermler *et al*. 2010, 2011, Orton *et al*. 2011, 2012, 2013) and retrieved information about human serum or lipid levels of AR antagonists from the peer-reviewed literature. We identified 22 chemicals for which both human tissue levels and concentration–response relationships for AR antagonism *in vitro* were available (Table 1) and assessed whether measurable combination effects are to be expected in the MDA-kb2 assay, when AR antagonists are combined at levels measured in human tissues.

## Materials and methods

### Chemicals

5α-Dihydrotestosterone (DHT, CAS# 521-18-6, >97% purity) was purchased from Steraloids Ltd, (London, UK) bisphenol-A (BPA, CAS# 80-05-7, >99%), *n*-butyl paraben (CAS# 94-26-8, >99%), *n*-propyl paraben (CAS# 94-13-3, >99%), perfluorooctane sulphonate (tetrabutylammonium salt) (PFOS, CAS# 111873-33-7, >95%), 2,2′,4,4′-tetrahydroxybenzophenone (benzophenone 2, BP2, CAS# 131-55-5), 2-hydroxy-4-methoxybenzophenone (benzophenone 3 (BP3), CAS# 131-57-7, 98%), butylated hydroxyanisole (CAS# 25013-16-5, >98.5%), butylated hydroxytoluene (CAS# 128-37-8, >99%), and benzo(α)pyrene (BaP, CAS# 50-32-8) were purchased from Sigma–Aldrich Co (Dorset, UK). Ethyl paraben (CAS# 120-47-8, 99%) and methyl paraben (CAS# 99-76-3, 99%) were obtained from Acros Organics (Loughborough, UK) and PCB 126 (CAS# 57465-28-8), hexahydrohexamethylcyclopentabenzopyran (galaxolide, HHCB, CAS# 1222-05-5) and 6-acetyl-1,1,2,4,4,7-hexamethyltetraline (tonalide, AHTN, CAS#1506-02-1) from LGC Promochem (Teddington, UK). 3-Benzylidene camphor (3-BC, CAS# 15087-24-8) was provided by Induchem AG (Volketswil, Switzerland), 2,2′,4,4′,6-pentabromodiphenyl ether (BDE100, CAS# 189084-64-8) by Cambridge Isotope Laboratories, Inc. (Ibstock, UK), *p*,*p* *′*-dichlorodiphenyldichloroethylene (*p*,*p* *′*-DDE, CAS# 72-55-9) by Greyhound Chromatography (Birkenhead, UK) and 4-methylenbenzylidene camphor (4-MBC, CAS# 36861-47-9) by Merck & Co (Hoddeston, UK).

PCB 118 (CAS# 31508-00-6) was obtained from Ultra Scientific (Teddington, UK). 2,2′,3,4,4′,5′-Hexachlorobiphenyl (PCB 138, CAS# 35065-28-2), 2,2′,4,4′,5,5′-hexachlorobiphenyl (PCB 153, CAS# 35065-27-1) and 2,2′,3,4,4′,5,5′-heptachlorobiphenyl (PCB 180, CAS# 35065-29-3) were purchased from Riedel-de-Haen (Dorset, UK). Stock solutions were made by dissolving the compounds in ethanol (≥99.7%, VWR International Ltd, Lutterworth, UK). Stock solutions and all dilution series were stored at −20 °C. All other reagents were obtained from Sigma–Aldrich Co. or Invitrogen (Paisley, UK).

### Cell culture and (anti-)androgenicity assay

MDA-kb2 cells with the MMTV.luciferase.neo reporter gene construct (Wilson *et al*. 2002) were routinely maintained in Leibowitz-15 (L-15) medium (Invitrogen) supplemented with 10% FCS (Invitrogen) at 37 °C without additional CO_2_. We employed a modified version of the original assay protocol by Wilson as described in Ermler *et al*. (2010, 2011). The androgen DHT (0.25 nmol/l) was used as a positive control and to establish a baseline for co-exposure for testing of AR antagonists.

### Statistical concentration–response analysis

Statistical dose response regression analyses were conducted according to the best-fit approach (Scholze *et al*. 2001), by independently fitting various non-linear regression models to the same data set and selecting the best-fitting model on the basis of a statistical goodness-of-fit criterion, as described earlier (Ermler *et al*. 2010, 2011).

### Calculation of mixture effect predictions

**Figure 2:**
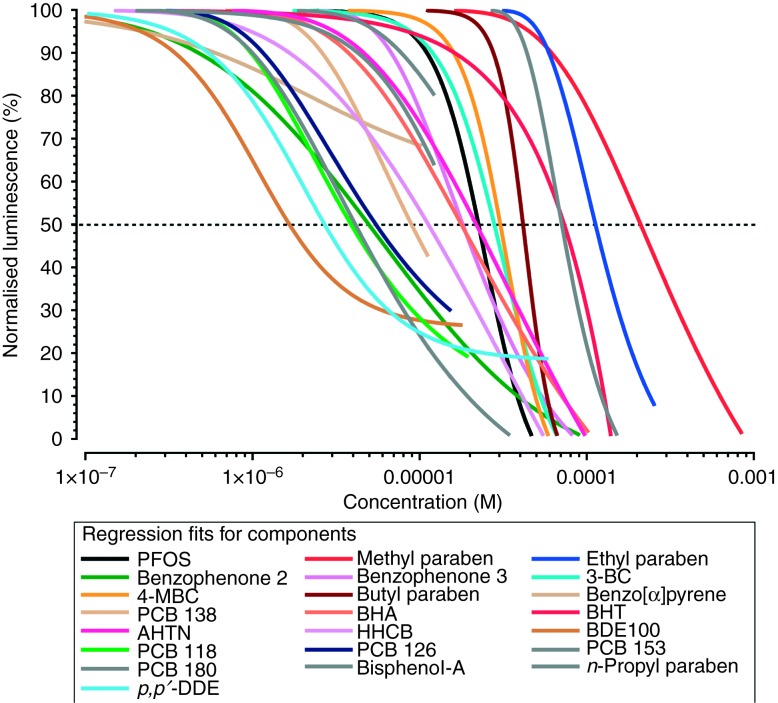
Concentration–response relationships for 22 AR antagonistic chemicals in the MDA-kb2 assay. Cells were exposed to increasing concentrations of the tested chemicals in the presence of 0.25 nM dihydrotestosterone (DHT). Shown are the best-fitting regression models.

Predictions for the combined effects of the test compounds were made by using concentration addition (CA), generally under the assumption of ‘non-interaction’ (i.e. each chemical in the mixture contributes to a combined effect, but without exacerbating or diminishing the effects of the other components). The mathematical and statistical procedures used for calculating mixture effects according to CA were the same as those described in Ermler *et al*. (2011) and Orton *et al*. (2012). The calculation of any effect concentration of a mixture under the hypothesis of CA was carried out using equation 1xref>.


(1)
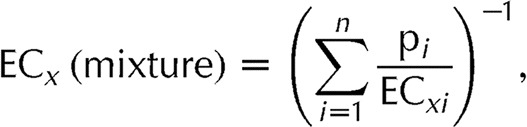



where EC_*xi*_ is the effect concentration of the *i*th compound in the mixture that on its own produces the same quantitative effect *x* as the mixture and p_*i*_ the relative proportion of the corresponding individual concentration present in the total mixture concentration. The individual effect concentrations were derived from the concentration–response functions for the compounds (Fig. 2 and Supplementary Table 1, see section on supplementary data given at the end of this article) by using their inverse functional form. The values for p_*i*_ were developed from human tissue levels (Supplementary Table 2).

Owing to its mathematical features, the CA concept cannot be used to calculate the effect concentrations associated with mixture effects that exceed the maximal AR antagonistic effect of the least efficacious compound present in the mixture. In the case investigated here, this limitation was introduced by BaP which showed AR antagonistic effects of a magnitude not exceeding 70% of the effect seen with DHT, corresponding to an AR antagonistic effect of 30%. To construct CA prediction curves that covered the entire range of antagonistic effects, we assumed that BaP did not contribute to the overall mixture effect at effect concentrations beyond the 30% effect.

### Collection of tissue level data for AR antagonists and conversion to serum levels as molar concentrations

Data of human tissue levels of AR antagonistic chemicals were collected and converted to molar concentrations (Table 1), with an emphasis on European data. To construct average exposure scenarios, we used averaged values, but for the assessment of high exposure scenarios we employed the highest reported values. For the u.v.-filters benzophenone 2 and 4-methylbenzylidene camphor, tissue level data were not available. To bridge this gap, we assumed that tissue levels are similar to those of the related compounds benzophenone 3 and 3-BC. We converted human serum levels (ng/ml) to molar concentrations, but where only adipose tissue levels were reported, we used the conversion method described for *p,p* *′*-DDE by López-Cervantes *et al*. (2004) to arrive at estimated serum concentrations. Conversions of data from serum levels on a lipid basis (ng/g) to concentrations on a wet basis (ng/ml) were performed by dividing the levels by a factor of 129.8. Reported adipose tissue levels were further divided by a factor of 4.2 to obtain an estimate of the concentration on a lipid basis. These conversion factors were specifically validated for *p,p* *′*-DDE (López-Cervantes *et al*. 2004), but in the absence of similar validated conversion factors for the compounds considered here, we applied these factors generally to all the 22 selected chemicals.

## Results

For the 22 chemicals listed in Table 1, we recorded concentration–response relationships for AR antagonism in the MDA-kb2 gene reporter assay (Fig. 2). We utilised the regression models for these chemicals (Supplementary Table 1) to predict their combined additive effects (dose addition) in the MDA-kb2 assay, for two scenarios.

**Figure 3:**
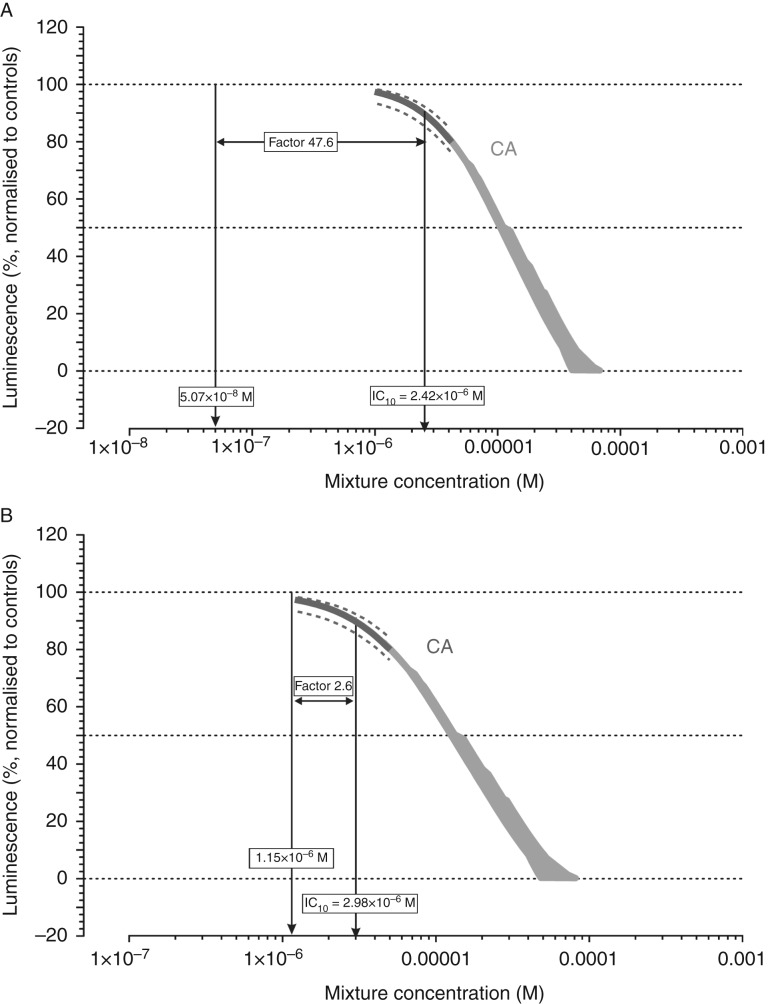
Predicted AR antagonistic effects in the MDA-kb2 assay for a mixture ration in proportion to average human serum levels of all 22 AR antagonists (A) and in proportion to high-end serum levels (B). The combination effects were calculated by using dose addition (dark grey line) with confidence belts (broken lines) The light grey belts depict predictions derived from extrapolations beyond the single effects of benzo(α)pyrene (for details see ‘Materials and methods’ section). The vertical arrows to the left indicate the sum of the concentrations of all 22 chemicals in serum, for the two investigated exposure scenarios. The arrows to the right depict the effect concentrations of the mixture predicted to be associated with a 10% AR antagonistic effect (IC_10_).

For the first scenario, we modelled average exposures. Accordingly, Fig. 3A shows the anticipated combination effects for a mixture with a mixture ratio equalling the average serum concentrations determined for each of the 22 chemicals and for multiples of these concentrations. AR antagonistic effects can be expected when the sum of the concentrations of all 22 components exceeds values higher than about 2.4 μmol/l, a concentration predicted to be associated with a 10% AR antagonistic effect, IC_10_ (for details of the mixture ratios refer to Supplementary Table 2). However, the sum of the average serum concentrations of all 22 compounds reaches only 50 nmol/l, which is by a factor of 48 lower than the predicted IC_10_ for the combination. AR antagonistic effects would therefore not become apparent in the MDA-kb2 assay, in which the 22 selected chemicals were tested at the average molar concentrations in human serum.

In the second scenario, we evaluated the expected joint effects for serum levels equivalent to high exposures. This time, the sum of serum concentrations of all compounds (1.1 μmol/l) was only by a factor of 2.6 lower than the total mixture concentration predicted to produce 10% AR antagonistic effects (ca. 3 μmol/l; Fig. 3B). With the statistical power afforded by the MDA-kb2 assay, which is sufficient to demonstrate the effect magnitudes of 10% (Ermler *et al*. 2010), this concentration would still be too low to produce measurable effects.

Previous mixture studies from our laboratory were intended to assess the predictability of combination effects (Ermler *et al*. 2011, Orton *et al*. 2012, 2013), and to achieve this, all chemicals had to be combined at mixture ratios proportional to their potency. In this so-called balanced mixture design, the components contribute in equal measure to the joint effect, because the toxic unit or risk unit in equation 1, i.e. the ratio of p_*i*_ (the relative proportion of the individual concentration of a component to the total mixture concentration) and EC_*xi*_ (the effect concentration of the *i*th compound in the mixture that on its own produces the same quantitative effect *x* as the mixture) have the same values for all mixture components.

**Figure 4:**
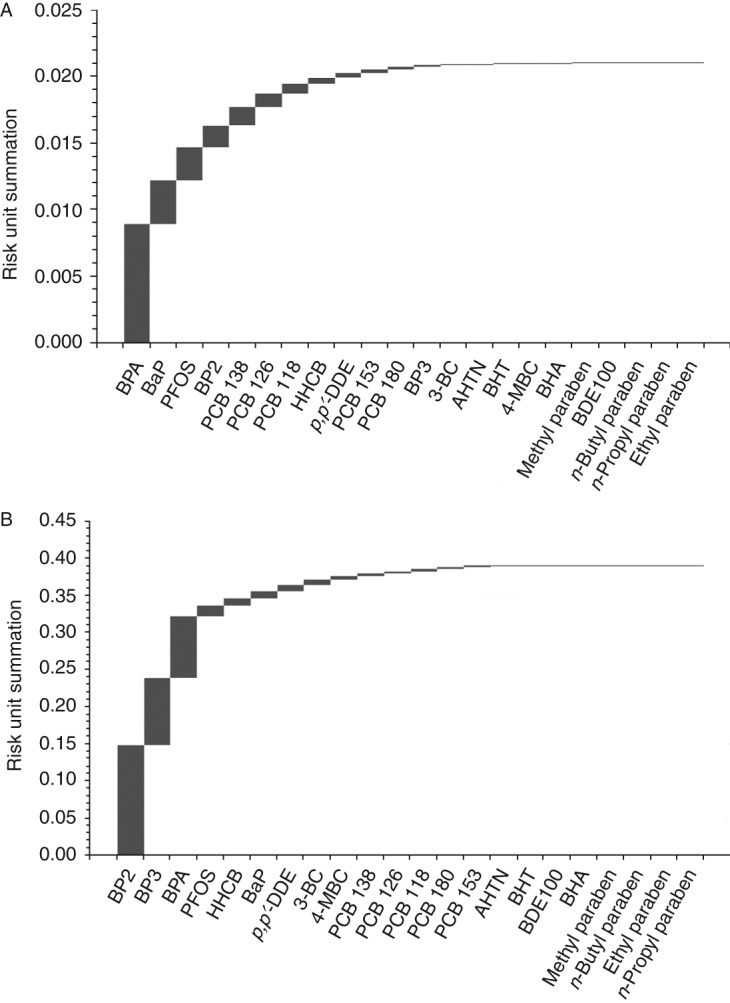
Cumulative risk unit summations for the two mixture predictions depicted in Fig. 2, for average human serum concentrations (A) and high-end levels (B).

However, in the two scenarios investigated here, this condition was not fulfilled, because the components were assumed to be present in proportion to their concentrations in body fluids. These concentrations are not correlated to the potency of the mixture components. It is therefore to be expected that some chemicals, due to their prevalence and potency, contribute disproportionately to the overall mixture effect, while the impact of others may be negligible. To visualise this for the two scenarios assessed here, we calculated the risk units for each component based on the concentrations present in a mixture expected to produce a 10% AR antagonistic effect (Supplementary Table 2). We then produced rank orders by arranging the risk units according to their magnitude and generated cumulative plots of the sums of risk units (Fig. 4).

For both the average and high exposure scenario, these plots showed that a relatively small number of mixture components explained the majority of the predicted combined effect. In the case of the average exposure scenario (Fig. 4A), the sum of the four largest risk units (0.016, from BPA, BaP, PFOS and benzophenone 2) already amounted to about 80% of the total sum of risk units (0.021). These four chemicals, together with those with the next four largest risk units (PCBs 138, 126, 118 and HHCB), explained 95% of the overall combined effect. Very similar results became apparent for the high exposure scenario (Fig. 4B), only that the rank order of the risk units changed. For high serum concentrations, benzophenone 2 and 3, BPA, PFOS, HHCB, BaP, 3-BC and 4-MBC together made up 95% of the total sum of risk units, while the PCBs and *p,p* *′*-DDE contributed very little to the combined effect in this scenario. These proportions are close to Pareto's rule, which states that ∼20% of the causal factors determine 80% of the effects.

## Discussion

### Interpretation of findings

Our calculations show that AR antagonistic combination effects demonstrable in the MDA-kb2 assay are not expected to occur when the selected 22 *in vitro* AR antagonists are combined at serum concentrations encountered in European countries, neither at levels representative of average exposures, nor at high exposures. It appears that the potency of the selected chemicals is too low to produce effects at serum levels, resulting from environmental exposures. Another way of interpreting our findings would be to say that we did not consider sufficiently large numbers of environmental antiandrogens to reach concentration ranges associated with effects.

### Constraints and limitations

This last aspect is a consequence of the constraints imposed by the lack of the data required to conduct modelling studies of the kind presented here. There are several hundred chemicals capable of antagonising the AR *in vitro* (Kojima *et al*. 2004, Vinggaard *et al*. 2008), but data about human tissue levels are available only for a small fraction of these substances. As pointed out earlier, this limitation is particularly relevant to the numerous pesticides identified as *in vitro* AR antagonists (Kojima *et al*. 2004, Vinggaard *et al*. 2008, Orton *et al*. 2011). For these reasons, we were unable to take account of a larger number of chemicals including pesticides.

A second limitation concerns the antiandrogenic modalities that could be incorporated into our study. Certain phthalates may contribute to androgen insufficiency in foetal life, not by blocking the AR as the agents investigated here, but by suppressing foetal androgen synthesis (Howdeshell *et al*. 2008). However, this mode of action cannot be captured in the MDA-kb2 assay and co-exposure to phthalates would have little impact on the endpoint measured in that assay. For this reason, we could not include phthalates in our calculations. Modelling of the joint effects of AR antagonists and phthalates would have required an integration of combined effects at a higher physiological level, measurable only in *in vivo* studies where it has been shown that certain phthalates and AR antagonists can work together to affect landmarks of male sexual differentiation in the rat (Christiansen *et al*. 2009, 2012). However, it is possible to take account of the impact of phthalates in a qualitative manner (see below).

### Defining conditions when combination effects are likely to occur

Despite these constraints and limitations, our efforts help to define conditions when combination effects of a sufficient magnitude might occur, by applying the principles of dose addition. We found that the sum of the average serum concentrations of our selected AR antagonists was lower by a factor of 48 than the total mixture concentration anticipated to produce a 10% AR antagonistic effect. To achieve an effect of such magnitude, there are two options, both in line with the principles of dose addition: one possibility is to increase the concentration of each of the 22 mixture components 48-fold (which is the option represented in Fig. 3A). Alternatively, if the concentrations of the chemicals are to be kept at the levels determined in serum, the only route open to reach the same effect is by increasing the number of AR antagonistic substances included in the mixture by a factor of 48. This would bring the number of mixture components to 1056 (=22×48). At present, this many AR antagonists are not known, and we have doubts whether such numbers will be identified in human tissues.

However, with the high exposure scenario, we found that the sum of the serum concentrations was only 2.6-fold lower than the mixture concentration needed to provoke effects. If we apply the same calculation to this case, 57 (=22×2.6) AR antagonists at levels equivalent to high-end serum concentrations will have to be combined to achieve a 10% AR antagonistic mixture effect. This number is closer to the number of chemicals with known *in vitro* AR antagonist properties. Based on these calculations, it can be expected that problematic situations are more likely to arise from high exposures, but not from average exposure scenarios.

### The impact of the magnitude of the AR antagonistic effect

We have based our predictions on a 10% AR antagonistic effect, a magnitude that can be captured with the statistical power afforded by the MDA-kb2 assay (Ermler *et al*. 2010). However, we wondered what the impact on the estimated numbers of mixture components would be if stronger antagonistic effects were demanded, for example 50%. As can be seen from Fig. 3, for both average and high exposures, the requirement to achieve a 50% antagonistic effect would increase the total mixture concentration to about 13 μmol/l, 256 or 11 times higher than the measured serum levels respectively. With the demand of keeping the concentrations the same as those of the 22 chemicals in each scenario, the required number of mixture components will then increase to 5632 (=22×256) in the case of average exposures, and 242 (=22×11) for high exposures, five or four times higher than the numbers necessary to achieve a 10% antagonistic effect. The impact of the required effect magnitude is largely driven by the gradient of the concentration response curve of the mixture, with steeper gradients lessening the impact of effect size.

### The implications of Pareto's rule

The cumulative risk unit summations depicted in Fig. 4 show that four (average exposures) or three (high exposures) of the 22 chemicals in the mixtures accounted for 80% of the sum of risk units. This is equivalent to 19% (average exposures) or 14% (high exposures) of the chemicals in the mixtures, close to Pareto's 20:80 rule. In both cases, more than half of the mixture components contributed minimally to the sum of risk units. These chemicals therefore represent ‘ballast’ that bulks out the estimated numbers of chemicals needed to provoke a combined effect. Although present in the mixtures, their influence is minimal, mainly because their potency is too low to make an impact at the measured serum levels. We note that some chemicals that constitute this ‘ballast’, especially the PCBs and *p,p* *′*-DDE, have been the focus of epidemiological studies exploring associations with TDS disorders. Strikingly, none of the AR antagonists contributing most to the mixture effect have been investigated epidemiologically. Application of Pareto's rule allows us to correct the above estimated numbers of chemicals downwards, to 211 (20% of 1056) and 11 (20% of 57) in the case of average and high-end exposures, respectively, needed to provoke a 10% antagonistic effect in the MDA-kb2 assay. In the case of an effect magnitude of 50%, these numbers reduce to 1126 (20% of 5632) or 48 (20% of 242) for average or high exposures respectively.

### Simplifying assumptions and their impact on our estimates

We had to base our calculations on a number of largely unproven assumptions which will be discussed here, in terms of their impact on our estimated antiandrogen numbers (for a summary see Table 2). Many of the chemicals considered here undergo conjugation reactions, yielding conjugates considered to have no biological activity. These reactions are particularly relevant to phenolic substances, such as BPA, parabens and similar chemicals. The literature we drew on to obtain information about human tissue levels of our chosen chemicals reported total serum or lipid levels, without distinguishing free parent compounds from their conjugates. For this reason, we could not take the impact of conjugation reactions into consideration but qualitatively, this is to be expected to reduce the combined effects of the mixtures and conversely, to increase the numbers of chemicals required to provoke AR antagonistic effects. Owing to a lack of appropriate data, we are at present unable to quantitate this impact. Similarly, it is difficult to factor in the influence of exposure duration on the strength on AR antagonistic effects. While exposures in the *in vitro* assay only last for 24 h, sensitive foetal tissues come into contact with the chemicals of interest for much longer periods of time.

As mentioned earlier, the impossibility of taking account of antiandrogens that exert their effects via modes of action different from AR antagonism may have led to underestimations of the number of chemicals needed to reach critical foetal levels. Without being able to quantify the impact of, for example phthalates, which lead to androgen insufficiency by suppressing androgen synthesis, their influence can at least be considered qualitatively: their presence will lead to a reduction in the critical number of AR antagonists.

An absence of data also forced us to make simplifying assumptions when constructing the high exposure scenario. The simple combination of high tissue levels, as done for our calculations, presupposes that high tissue levels of contaminants are correlated, i.e. that there are subjects who experience high exposures to all the 22 chemicals considered here. We acknowledge that this is a highly unlikely scenario, representative of a worst-case assumption. However, multiple pollutants are rarely measured in one and the same human tissue specimen, and we could not locate literature where the levels of all of our chosen chemicals were reported together, but such data would be needed to put the reconstruction of a high-end exposure scenario on a more solid footing. We suggest that this issue can be approached by conducting probabilistic analyses beyond the scope of this study. Nevertheless, it is clear that our simplifying assumptions regarding high exposures have driven upwards the anticipated mixture effects, and downwards the estimated number of chemicals.

### Implications for extrapolations to physiological scenarios relevant to humans

For the majority of the *in vitro* AR antagonists considered here, data about the ability to produce antiandrogenic effects *in vivo* is lacking entirely. For this reason, reflections about the physiological relevance of our analysis have to be undertaken with great care, and by making explicit all relevant assumptions. This exercise will expose factors that are currently undefined, but may have a great impact on the extent of antiandrogenic effects (see the compilation in Table 2).

First, let us apply the scenario investigated thus far to the tissue dosimetric approach taken by You *et al*. (1999) in their analysis of the antiandrogenic effects of *p,p*-DDE in the rat. By using the *in vitro* AR antagonistic data for *p,p* *′*-DDE reported by Wilson *et al*. (2002) and Kojima *et al*. (2004), it can be shown that antiandrogenic effects only became manifest when sufficiently high levels of *p,p* *′*-DDE had accumulated in the male foetus, levels clearly associated with *in vitro* AR antagonistic effects. In every likelihood, the dose addition principle also applies here: it should be possible to replace the biologically active internal foetal dose of *p,p* *′*-DDE with equi-effective fractions of several other AR antagonists, without loss of effect. Whether this is achievable in the rat (let alone the human) with the chemicals investigated here is largely unexplored, but should depend primarily on toxicokinetic factors. It is quite conceivable that the doses of certain *in vitro* active compounds that have to be administered singly to pregnant dams to attain foetal AR antagonistic tissue levels are so high that maternal toxicity is induced, in which case the *in vitro* activity of such chemicals will be irrelevant because it cannot be expressed *in vivo*. Nevertheless, in the light of the available evidence of the behaviour of *in vitro* AR antagonists in combination (Birkhoj *et al*. 2004, Kjærstad *et al*. 2010, Ermler *et al*. 2011, Orton *et al*. 2012, 2013), there is no reason to believe that the 22 chemicals investigated here should not cause antiandrogenicity in the rat if they were present in the foetus at the concentrations shown to produce AR antagonistic effects *in vitro* in the MDA-kb2 assay. Viewed from the perspective of tissue dosimetry, *in vitro*–*in vivo* extrapolations lose much of the mystique that is often attached to them.

If we continue our thought experiment by considering the rat, the next question to be examined concerns the relationship between serum levels of AR antagonists and those in foetal tissues. Again, these relationships are largely unexplored, but it would be plausible to assume that the two concentrations are not drastically different, unless there are processes that lead to the accumulation in foetal tissues of certain chemicals. The blood–placenta barrier is another factor to consider, but many chemicals evaluated here are able to cross this barrier.

It remains to reflect on the critical AR antagonistic effect magnitude that a foetus is unable to tolerate without suffering androgen insufficiency. It is presently unknown whether this is equivalent to a 10% effect or closer to 50%, and will not be known in the foreseeable future. However, as discussed above, the likely impact depends on the gradient of the underlying dose–response curves.

Finally, we need to deliberate on the question of the sensitivity of the human foetus relative to that of the rat foetus. These sensitivity differences are currently unknown, but certain assumptions can be made and their impact can be elaborated. The sensitivity of the foetus is the factor likely to have the largest impact on the number of antiandrogens that need to be invoked to make a case for potentially adverse tissue concentrations. If the sensitivity of foetal tissues is by a factor of only 10 higher than the sensitivity of an *in vitro* assay with MDA-kb2 cells, the predicted dose additive curves in Fig. 3 will shift by one order of magnitude towards lower concentrations. In the case of the high exposure scenario investigated here, this would mean that the 22 *in vitro* AR antagonists together would already show joint effects if they were present at these levels in the foetus. Whether this is realistic is at present difficult to judge, but an answer to this question will depend on better information about the relative sensitivities of rat and human foetuses to AR antagonists. Similar considerations apply to subgroups of the population with enhanced sensitivity to AR antagonists.

### Is there an explanation gap?

Our analysis forces the conclusion that rampant TDS disorders cannot at present be explained in terms of average human exposures to 22 AR antagonists known to be present in human tissues. Their potencies and their tissue levels are too low to attain AR antagonistic effects of a critical magnitude. Conversely, the number of AR antagonists that one would need to invoke, under the assumption that both their tissue levels and their potencies are comparable to those of known AR antagonists present in human tissues, reaches 250–5500 chemicals. The upper estimate even exceeds the 2000 *in vitro* AR antagonists estimated on the basis of quantitative structure–activity relationships to be in commercial use (Vinggaard *et al*. 2008).

The picture that emerges in the case of high exposures is not as clear-cut. Here, between 10 and ∼250 chemicals with potencies and tissue levels comparable to known AR antagonists would be required to attain critical effects, and clearly these numbers seem to be more realistic in relation to the number of known AR antagonists. But even under this scenario, there is currently an explanation gap because the lower estimate of about ten chemicals applies to agents that make a large contribution to a joint effect, according to the Pareto principle. The identity of such chemicals is currently not fully known.

It would appear that these explanation gaps can only be bridged by conjuring up as yet undiscovered high-potency AR antagonists, or alternatively high exposures to unknown agents of average potency. Furthermore, it is clear that it will be difficult to explain TDS disorders solely on the basis of AR antagonists. Other antiandrogenic modalities such as suppression of foetal androgen synthesis (e.g. by phthalates) will also need to be taken into account. But even if we consider joint effects between phthalates and known AR antagonists, critical effect magnitudes may be hard to reach.

With the realisation of the possible human health consequences of suppressing prostaglandin synthesis in foetal life, an additional ‘antiandrogenic’ modality has recently become the focus of attention. Four epidemiological studies have shown that the use of paracetamol (acetaminophen) and other analgesics in pregnancy is associated with an increased risk of cryptorchidism in boys (Berkowitz & Lapinski 1996, Jensen *et al*. 2010, Kristensen *et al*. 2011*a*, Snijder *et al*. 2012), and exposure to these drugs is surprisingly high. Paracetamol (acetaminophen) and aspirin were shown to induce antiandrogenic effects by a mechanism involving suppression of prostaglandins, agents important for male sexual differentiation (Kristensen *et al*. 2011*a*). A variety of other endocrine disrupting chemicals have been identified as being capable of suppressing prostaglandin synthesis *in vitro* (Kristensen *et al*. 2011*b*). It remains to be seen whether consideration of this new antiandrogenic modality might help to bridge the explanation gaps that we exposed here, but we note that *in vitro* potencies of many prostaglandin-suppressing endocrine disrupters are higher than those reported for AR antagonists.

It will be necessary to search for, and identify, new antiandrogenic chemicals that are present in human tissues in an ‘exposome’ approach. This requires systematic and concerted efforts, an area of research that has been neglected in the past, very much to the detriment of environmental epidemiology. Stephen Rappaport's lament (Rappaport *et al*. 2012) of the state of environmental epidemiology also applies to antiandrogens and the search for explanations of TDS disorders: ‘… with few exceptions, the identities of major environmental toxicants and their roles in causing chronic disease have not been addressed. Given the poor state of knowledge about health-impairing environmental exposures, epidemiologists pursue narrow hypotheses that largely skirt disease aetiology in favour of known environmental risk factors even when the attributable risks are small. Although such hypothesis-driven studies confirm some environmental sources of disease, they offer only fragments of our understanding of the major causes and mechanisms of chronic diseases.’

## Discussion from meeting

**Philippe Grandjean** (Odense, Denmark): Your provocative presentation is very interesting and your conclusions might be valid, but would be more convincing if you built in a degree of susceptibility. Thus, vulnerability should also be included in your Monte Carlo model. You modelled the sensitivity to androgen receptor antagonists as being tenfold higher *in utero* than suggested by *in vitro* assays, but there are possibly also a variety of factors, particularly on as yet unknown genetic predisposition, which must be present to explain the gap. This is a variable which you must consider to determine if genetic predisposition rather than the number of chemicals determines the outcome.

**A Kortenkamp** (Uxbridge, UK): That is a good suggestion. Rather than modelling, we are trying to define a framework which will isolate factors that will make a big difference from those which do not have such a strong impact. The sensitivity, vulnerability and predisposition of the subjects will have a massive impact on the number of chemicals we have to invoke. In this context, the magnitude of the effect is not as important.

**Louis Guillette** (Charleston, USA): It has been emphasised at this meeting that mixtures are important. Studies in humans, wildlife and laboratory animals show that multiple endocrine activities are taking place simultaneously. Earl Gray has examined multiple modalities with antiandrogenic activity and has described ‘something from nothing’ effects due to the summation of minor events. You should consider what modalities other than antiandrogenic substances could have oestrogenic activity. Glucocorticoid signalling might also be involved. Michael Eisenberg (this volume) described many isoforms of the androgen receptor (AR). Most laboratory tests are targeted on single clones, but there is a marked diversity in the AR in the human population especially between different ethnic populations.

**A Kortenkamp**: We have conducted various AR *in vitro* antagonist assays and find that the potency is very comparable with agreement between different assays. Modalities other than AR antagonism are difficult to model because it is not clear how these different modalities result in common mixture effects at a level of biological organisation further removed from interaction with receptors. We have taken account of this by qualifying our estimates with strong provisos. There is currently no other way of dealing with this.

**Shanna Swan** (New York, USA): There are many non-chemical endocrine disrupters such as stress, nutrition, sleep and lack of exercise. We have seen that in low stress mothers there is a twofold increased susceptibility to phthalates in relation to the anogenital distance (AGD) of the offspring. Some of these multiple non-chemical factors can act as antiandrogens.

**A Kortenkamp**: Your point is much appreciated. These non-chemical factors probably act by altering sensitivity to chemical compounds and can be taken into account in our framework by making different assumptions about the vulnerability of the foetus.

**Anne Marie Vinggaard** (Søborg, Denmark): Your estimations of the hazard index might not be valid because these are based only on AR antagonism. You have not included chemicals which inhibit testosterone production and these have an additive effect to AR antagonism. Also, the IC_50_ or IC_10_ values might not be appropriate for your calculations because they fail to take account of protein binding when extrapolating from *in vitro* to the *in vivo* situation. For example, the effect of PAHs has been shown to be more potent *in vivo* if protein binding *in vitro* is considered.

**A Kortenkamp**: I agree that other modalities are relevant to the disorders that we see, but it is difficult to take these into account with our tentative modelling approach other than by stating the direction in which the presence of phthalates and other chemicals with different modes of action will affect our estimates. They will likely reduce the number of chemicals we need to invoke. That is currently the only way to take account of the factors you mentioned because it is impossible to aggregate the effects of phthalates and AR antagonists at the tissue level, where we measure the concentrations in tissue and at the cellular level. Animal models are required to explore the combined effect.

**Greet Schoeters** (Mol, Belgium): You complicate matters by studying mixtures rather than single compounds, but the outcome of your *in vitro* assay addresses a single target. Endocrine disrupters cause syndromes such as testicular dysgenesis syndrome (TDS), which are a mixture of different events. How does this fit into your model?

**A Kortenkamp**: We were investigating the magnitude of the problem by considering mixture effects. It will be near impossible to explain current trends in male reproductive health without taking account of mixtures. In order to make progress, we had to simplify matters somewhat by making sometimes quite crude assumptions.

**Jerry Heindel** (NIEHS, USA): How many mixtures have you tested and are you sure you are correct? If you predict that a mixture will have no effect, have you tested the mixture to make sure?

**A Kortenkamp**: We have performed the experiments and found that mixtures had no effect as calculated.

## Supplementary data

This is linked to the online version of the paper at http://dx.doi.org/10.1530/REP-13-0440.

## Figures and Tables

**Table 1:** Tissue levels of each compound for an average and a high exposure scenario.

**Compounds** (in order of EC_50_)	**Average levels** (M)	**High levels** (M)	**References**
Polybrominated diphenyl ether 100	8.12×10^−13^	1.59×10^−11^	Meneses *et al*. (1999), Covaci *et al*. (2002) and Ramos *et al*. (2007)
Benzophenone 2	7.50×10^−10^ ^a^	7.00×10^−8^ a,b	–^a^
Polychlorinated biphenyl 118	7.08×10^−10^	2.83×10^−9^	Koppen *et al*. (2002) and Park *et al*. (2007)
*p,p* *′*-DDE	1.90×10^−10^	4.70×10^−9^	Galassi *et al*. (2008)
Benzo(α)pyrene	1.98×10^−9^	5.55×10^−9^	Neal *et al*. (2008)
Bisphenol-A	8.76×10^−9^	8.28×10^−8^	Schoenfelder *et al*. (2002) and Vandenberg *et al*. (2007)
Polychlorinated biphenyl 126	1.18×10^−9^	3.54×10^−9^	Koppen *et al*. (2002)
Galaxolide (HHCB)	7.10×10^−10^	1.59×10^−8^	Hutter *et al*. (2005) and Schiavone *et al*. (2010)
Polychlorinated biphenyl 138	3.63×10^−9^	8.75×10^−9^	Koppen *et al*. (2002) and Park *et al*. (2007)
Butylated hydroxyanisole	5.09×10^−11^	1.02×10^−10^	Conacher *et al*. (1986)
Polychlorinated biphenyl 180	9.74×10^−10^	1.21×10^−8^	Koppen *et al*. (2002) and Park *et al*. (2007)
Tonalide (AHTN)	1.77×10^−10^	3.10×10^−9^	Hutter *et al*. (2005) and Schiavone *et al*. (2010)
Benzophenone 3	8.09×10^−10^	6.00×10^−7^ ^b^	Hany & Nagel (1995) and Schlumpf *et al*. (2010)
Polychlorinated biphenyl 153	2.13×10^−9^	1.67×10^−8^	Koppen *et al*. (2002) and Park *et al*. (2007)
Perfluorooctane sulphonate	2.70×10^−8^	1.56×10^−7^	Kannan *et al*. (2004), Midasch *et al*. (2006), Fromme *et al*. (2007) and Karrman *et al*. (2007*a*, 2007*b*)
3-Benzylidene camphor	4.81×10^−10^ ^a^	8.32×10^−8^ ^a^	–^a^
4-Methylbenzylidene camphor	4.54×10^−10^	7.86×10^−8^	Janjua *et al*. (2004) and Schlumpf *et al*. (2010)
Butylated hydroxytoluene	4.99×10^−10^	9.99×10^−10^	Conacher *et al*. (1986)
*n*-Butyl paraben	2.46×10^−11^	1.09×10^−10^	Darbre *et al*. (2004)
*n*-Propyl paraben	2.34×10^−11^	2.34×10^−11^	Darbre *et al*. (2004)
Ethyl paraben	2.21×10^−11^	8.17×10^−11^	Darbre *et al*. (2004)
Methyl paraben	1.54×10^−10^	3.53×10^−10^	Darbre *et al*. (2004)

a Adjusted values to avoid domination of the mixture effect.

b No direct tissue levels available, thus the values were estimated from intake levels, compared with related compounds (BP2 with BP3 and 4-MBC with 3-BC).

**Table 2:** Assumptions that are currently difficult to verify but which have an impact on the estimated number of AR antagonists necessary to yield critical effects.

**Assumption**	**Impact on estimated numbers of AR antagonists required to reach critical effects**
Critical effect magnitude of AR antagonistic effects larger than 10%	Will increase
Conjugation reactions remove biologically active AR antagonists	Will increase
Exposure duration of foetal tissues is longer than in the AR antagonist assay	Longer exposure times may increase the effectiveness of AR antagonists, and decrease the number of chemicals needed to reach critical effects
Only AR antagonists could be considered	Consideration of other antiandrogen modalities will decrease the critical number of chemicals
High internal exposures are correlated	If high exposures to chemicals are not correlated, larger numbers of AR antagonists will be required to reach critical effects
Serum levels of AR antagonists are similar to those in foetal tissues	Numbers will increase if foetal levels are lower
Sensitivity of human foetal tissues is comparable to that of the rat	If the human is more sensitive, estimates of numbers of chemicals to reach critical effects will decrease
Mutations and similar factors predispose subgroups of the population to greater sensitivity to AR antagonists	Critical number of AR antagonists will decrease
